# Comparison of patency of single and sequential radial artery grafting in coronary artery bypass

**DOI:** 10.1093/icvts/ivab279

**Published:** 2021-10-23

**Authors:** Hirofumi Kasahara, Hankei Shin, Tatsuo Takahashi, Satoru Murata, Mitsuharu Mori

**Affiliations:** 1 Department of Cardiovascular Surgery, Tokyo Dental College Ichikawa General Hospital, Chiba, Japan; 2 Department of Cardiovascular Surgery, Keio University School of Medicine, Tokyo, Japan; 3 Department of Cardiovascular Surgery, Saiseikai Utsunomiya Hospital, Tochigi, Japan

**Keywords:** Radial artery, Coronary artery bypass grafting, Sequential grafting, Anastomotic patency, Graft patency

## Abstract

**OBJECTIVES:**

Sequential radial artery (RA) grafting has the potential to enhance arterial revascularization compared to single grafting. Sequential RA grafting was performed predominantly with a single side-to-side anastomosis. The study aimed to assess if sequential RA grafting improved long-term graft patency compared to single RA grafting. In addition, the anastomotic patencies of side-to-side and end-to-side anastomoses in sequential RA grafting were assessed.

**METHODS:**

Two hundred nineteen patients underwent isolated coronary artery bypass grafting with skeletonized RA conduits between 2005 and 2016. Of these, 208 patients underwent radiological graft assessment; thus, 125 and 83 patients underwent single and sequential RA grafting, respectively. The graft and anastomotic patency rates were estimated using the Kaplan–Meier method.

**RESULTS:**

The median follow-up period was 9.1 years, and the radiological assessment lasted 5.1 years. The overall RA graft patency rates at 1, 5 and 10 years were 99.4%, 92.7% and 88.1%, respectively. The RA graft patency rate for sequential grafting was similar to that for single grafting (88.7% vs 87.4% at 10 years; *P* = 0.88). In the stratified analysis of anastomotic patency, the patency rate of side-to-side anastomoses of sequential RA grafting was significantly better than that of end-to-side anastomoses (100% vs 88.7% at 10 years; *P* = 0.01).

**CONCLUSIONS:**

The long-term RA graft patencies of sequential and single grafting were equally high. The anastomotic patency of side-to-side anastomoses of sequential RA grafting was remarkably high. Considering these findings, the RA can be effectively used for multiple arterial coronary revascularizations.

**Figure:**
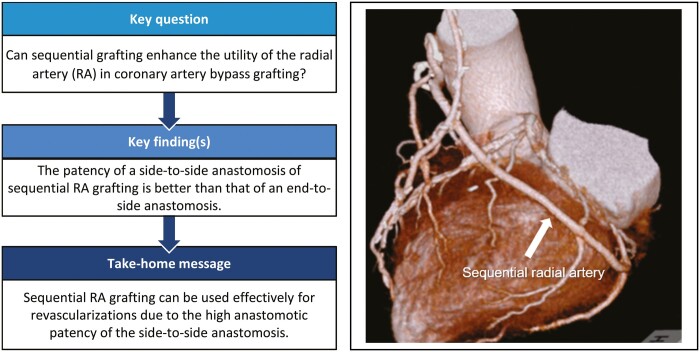

## INTRODUCTION

Coronary artery bypass grafting (CABG) is a major procedure for improving survival in patients with obstructive coronary artery disease. Grafting of the left internal thoracic artery (ITA) to the left anterior descending artery (LAD) is recognized as the gold standard due to the high long-term graft patency and improvement in late survival [1]. CABG using bilateral ITAs improves long-term survival when compared to that using single ITAs [2, 3]; however, deep sternal infection when using bilateral ITAs remains concerning, especially in obese or diabetic patients [4, 5].

Carpentier *et al.* [6] introduced the radial artery (RA) as an arterial conduit in 1973. Harvesting and anastomosing of the RA are easier than that of the ITA due to the relatively thicker wall and greater diameter. The length of the RA is usually enough for sequential grafting [7]. In recent randomized controlled trials comparing the RA and the saphenous vein (SV) as the second graft, a significant reduction in cardiac events and higher graft patency with the RA were reported at 5 years [8]. The RAPCO trials (Radial Artery Patency and Clinical Outcomes) showed that the RA has superior patency to the free right ITA at 10 years [9].

Various reports have examined sequential venous grafting [10, 11]; however, reports regarding long-term sequential RA grafting are limited, and the effective use of RA grafting remains unclear. The present study investigated the early- and long-term outcomes of CABG with the RA, particularly whether sequential RA grafting improved graft patency compared with single RA grafting. In addition, we investigated whether the anastomotic patency of the side-to-side anastomosis is superior to that of the end-to-side anastomosis in sequential RA grafting.

## PATIENTS AND METHODS

### Design and patients

This retrospective study was approved by the Institutional Review Board of Tokyo Dental College Ichikawa General Hospital (I18-37); the need for written informed consent from patients was waived due to the retrospective study design. During 2005–2016, 618 patients underwent isolated CABG at our hospital and RA was used in 219 patients (35.4%). Of these, 208 patients who had radiological graft assessments were included in this study. To evaluate the usefulness of our sequential grafting strategy, the patients were divided into 2 groups: single and sequential RAs. Medical data were collected through a review of medical charts and our department database, including patient’s characteristics, comorbidities, surgical details, postoperative radiological graft assessment and postoperative complications. The patients were followed up regularly at our hospital or affiliated hospitals. Telephonic interviews and inquiries with family doctors were conducted in 12 patients who did not have recent follow-up visits to confirm their current status.

### Operative technique and grafting strategy

All operations were performed by the same surgical team, and most operations were performed by the chief surgeon (H.S.). The RAs were harvested in the non-dominant forearm with an open technique. The RA was harvested in a skeletonized manner. Connective tissues around the adventitia of the RA were dissected with scissors, leading to adequate dilatation. Small branches without satellite veins were cut with protein coagulation by an ultrasonic scalpel (Ethicon Endo-Surgery, Cincinnati, OH) or metal clips [12] (see Supplementary Material, Fig. S1). The harvested RA was wrapped with gauze soaked in diluted papaverine solution to eliminate vasospasm. CABG was performed using cold blood cardioplegic arrest in most cases. In-situ left ITA was anastomosed with the LAD in most cases; the RA was used mainly as the second and/or third graft. The RA graft was anastomosed to the coronary artery with luminal stenosis of 75% or more using 7–0 polypropylene suturing. The SV was used for complete re-vascularization in most cases. In sequential RA grafting, the RA was usually anastomosed to the target vessels transversely (diamond shape) to reduce the graft length and to prevent graft kinking. The distal end of the RA was anastomosed to a target vessel with a good run-off to prevent competitive flow. Sequential RA grafting with 1 side-to-side anastomosis was also employed as a grafting strategy; almost all patients, with the exception of 2, underwent sequential RA grafting according to this strategy. The proximal end of the RA was anastomosed directly to the ascending aorta with 6–0 polypropylene suturing after opening with a 4.0-mm aortic punch. Preoperative computed tomography (CT) scanning was routinely performed in all elective cases to evaluate ascending aortic atherosclerosis. Intraoperative epiaortic ultrasound scanning was applied at the surgeon's discretion.

### Graft and anastomotic assessments

Postoperative radiological graft patency and anastomotic patency were assessed by using a high-resolution 320-slice multidetector computed tomography (MDCT) angiography in most of the cases; 64-slice MDCT was used in the early cases. CT angiography was performed within 1 month after the operation. Thereafter, CT angiography was planned once every 4–6 years as a guide, but was performed at varying time-points. Moreover, the interval period and decision to perform the imaging study varied considerably among patients, depending on age, renal function and comorbidities. The CT angiograms were assessed by 2 independent specialists for coronary disease. In the case of conflict between the 2 reviewers, a third reviewer assessed the angiograms. Symptom-driven invasive coronary angiography was performed when indicated (*n* = 13), and the angiograms were assessed by several cardiologists.

The graft status was classified according to the Fitzgibbon classification [13]. FitzGibbon A is an excellent graft, FitzGibbon B is an impaired graft with stenosis or a string sing and FitzGibbon O is an occlusion graft. The use of FitzGibbon B or O or poor visibility of the conduit was defined to indicate graft failure in this study. For graft patency in sequential grafting, graft failure was considered if 1 anastomosis was FitzGibbon B or O or if the conduit was poorly visible, irrespective of side-to-side or end-to-side anastomosis.

For the stratified analysis of anastomotic patency, the coronary anastomoses in sequential RA grafting were counted separately, and the anastomoses were divided into 3 groups: side-to-side anastomoses in sequential RA grafting, end-to-side anastomoses in sequential RA grafting and end-to-side anastomoses in single RA grafting. Anastomotic patencies were compared in sequential RA grafts (between side-to-side and end-to-side) and among the 3 groups.

### Postoperative lipid and drug management

All patients received lifelong aspirin (100 or 200 mg daily) administered within 6 h after the operation. Intravenous administration of diltiazem hydrochloride (0.5–1.0 μg/kg/min) was started at the beginning of surgery and was followed by a lifelong oral intake of 200 mg daily. Diltiazem hydrochloride was changed to nicorandil in patients who developed bradycardia. Statin was strictly administered to maintain a low-density lipoprotein cholesterol (LDL-C) level below 100 mg/dl. Beta-blockers were also administered. These medications were continued unless they became contraindicated.

### End points

The primary end-point was graft patency. The secondary end-point was all-cause mortality.

### Pre- and postoperative variables

Diabetes, hypertension and hyperlipidaemia were identified using the current medical records. Cerebrovascular disease was considered to be present in patients with a history of stroke, transient ischaemic attacks or carotid artery intervention. LDL-C was measured just before the operation, approximately 1 month after CABG, and at least once every year.

### Statistical methods

Data were expressed as mean ± standard deviation (SD) when normally distributed or median with interquartile range when not normally distributed. *P*-values <0.05 were considered statistically significant. The Kaplan–Meier method was used to analyse all-cause mortality and graft occlusion. Subgroups were compared using log-rank tests. Categorical variables were compared using chi-squared or Fisher exact tests, as appropriate. Anastomoses within the same patient were considered statistically independent in the stratified analysis for anastomotic patency (see Graft and Anastomotic Assessments section). Statistical analyses were performed using IBM SPSS Statistics for Windows, Version 26.0 (Armonk, NY, USA).

## RESULTS

### Demographic and preoperative variables

The mean age of the 208 patients at the time of operation was 65 ± 9 years (range, 41–83 years). Overall, 208 patients underwent radiological graft assessment; 125 (60.1%) and 83 (39.9%) underwent single and sequential RA grafting, respectively. Table 1 summarizes the comparisons of the preoperative characteristics of the single and sequential RA groups. There were no differences in the patient characteristics with the exception of sex and use of off-pump bypass; there were fewer females (7.2% vs 22.4%, *P* = 0.004), and fewer patients underwent off-pump bypass (4.8% vs 16%, *P* = 0.01) in the sequential group (Table 1). No patient in either group had undergone prior CABG.

**Table 1:** Demographic characteristics of all patients based on whether a single or sequential radial artery graft was used

	Single RA *n* = 125	Sequential RA *n* = 83	P-value
Age	65.6 ± 9	63.7 ± 8.5	0.13
Sex (males)	97 (77.6)	77 (92.8)	0.004*
BMI	24.7 ± 3.4	24.8 ± 3.1	0.79
CCS	2.6 ± 0.8	2.7 ± 0.8	0.67
NYHA	1.7 ± 0.8	1.7 ± 0.9	0.8
Hyperlipidaemia	101 (80.8)	70 (84.3)	0.46
Preoperative statin	99 (79.2)	71 (85.5)	0.46
Hypertension	99 (79.2)	73 (88)	0.1
Diabetes mellitus	60 (48)	48 (56.4)	0.25
Insulin dependent	17 (13.6)	11 (12.9)	0.95
Smoking history	46 (36.8)	38 (45.8)	0.2
Obesity	36 (28.8)	29 (34.9)	0.35
Cerebrovascular disease	31 (24.8)	19 (22.9)	0.9
Previous PCI	37 (29.6)	17 (20.5)	0.14
Ejection fraction <40%	22 (17.6)	12 (14.5)	0.55
Peripheral arterial disease	11 (8.8)	3 (3.6)	0.17
Urgent operation	20 (16)	19 (22.9)	0.21
Emergency operation	14 (11.2)	10 (12)	0.85
IABP initiated before op	7 (5.6)	9 (10.8)	0.17
OPCAB	20 (16)	4 (4.8)	0.01*
Estimated GFR	68.1 ± 19	68.9 ± 18.5	0.77
Ejection fraction (%)	49.3 ± 15	52.7 ± 15.4	0.19
Observation period (months)	46.4 ± 38.6	55 ± 41.3	0.13

Data are presented as numbers and proportions or means ± standard deviations.

BMI: body mass index; CCS: Canadian Cardiovascular Society grading of angina pectoris; D: diagonal; GFR: glomerular filtration rate (ml/min/1.73 m^2^); IABP: intra-aortic balloon pumping; IM: intermediate; LAD: left anterior descending artery; LCx: left circumflex; NYHA: New York Heart Association functional classification; op: operation; OPCAB: off-pump coronary artery bypass grafting; PCI: percutaneous coronary intervention; RA: radial artery; RCA: right coronary artery.

* Statistical significance.

### Operative data

The in-hospital mortality rate was 0.5%. Complete revascularization was achieved in all patients. The total number of distal anastomoses was 822. The mean number of distal anastomoses per patient was 4.0 ± 1.3. Bilateral ITAs were used in 21 patients; 11 patients did not receive any ITA. The right gastroepiploic artery was used in 1 patient. The rate of total arterial revascularization was 17.8% (37 patients). The distribution of coronary anastomosis of RA grafts in each group is shown in Table 2. Supplementary Material, Table S1 shows the breakdown of all grafts used, including the number of anastomoses and anastomosis sites. Table 3 shows the target coronary artery stenosis for each graft, including the RA, ITA and SV.

**Table 2:** Distribution for coronary anastomosis of radial artery grafts

Distal anastomosis site	Single RA *n* = 125	Sequential RA *n* = 168	*P* = 0.2
RCA	25 (20)	7 (4.2)	
Right ventricular branch		1	
Segment 3	3		
Posterior descending	19	4	
Posterolateral branch	3	2	
LAD	4 (3.2)	7 (4.2)	
D, IM	20 (16)	79 (47)	
LCx	76 (60.8)	75 (44.6)	

D: diagonal; IM: intermediate; LAD: left anterior descending artery; LCx: left circumflex; RA: radial artery; RCA: right coronary artery.

**Table 3:** Anastomotic sites of the target vessels and the degree of coronary artery stenosis

	% stenosis	RA	ITA	SV
RCA		32	4	199
	50			5
	75	7	1	48
	90	15	2	60
	99	3	1	20
	100	7	0	66
LAD		11	204	
	50		5	
	75	1	83	
	90	5	63	
	99	1	15	
	100	4	38	
D, IM		99	24	18
	50			1
	75	34	7	4
	90	52	16	9
	99	8	0	1
	100	5	1	3
LCx		151	14	65
	75	44	4	12
	90	69	5	27
	99	16	3	9
	100	22	2	17
Total		293	246	282

D: diagonal; IM: intermediate; ITA: internal thoracic artery (left and right sides); LAD: left anterior descending artery; LCx: left circumflex; RA: radial artery; RCA: right coronary artery; SV: saphenous vein.

### Primary analysis: graft patency

Overall, 293 RA anastomoses of 208 patients were assessed using CT angiography within 1 month after the operation and 226 RA anastomoses of 166 patients were assessed in the late phase at least once. Forty-two patients (20.2%) had a CT assessment only early after surgery. There were 34 patients (16.3%) who underwent radiological evaluation once in the chronic phase but were not classified to undergo a complete radiological follow-up. In contrast, there were 132 patients (63.5%) classified as having almost complete radiological follow-up. Therefore, the median overall follow-up period for radiological assessment was 5.1 years (interquartile range, 1.9–7.3) (*n* = 208). The median angiographic late phase follow-up period was 6.2 years (interquartile range, 3.9–8) (range 1–14) after CABG (*n* = 166). Patency of 246 ITA anastomoses was also assessed simultaneously (see Supplementary Material, Table S1).

**Figure 1:**
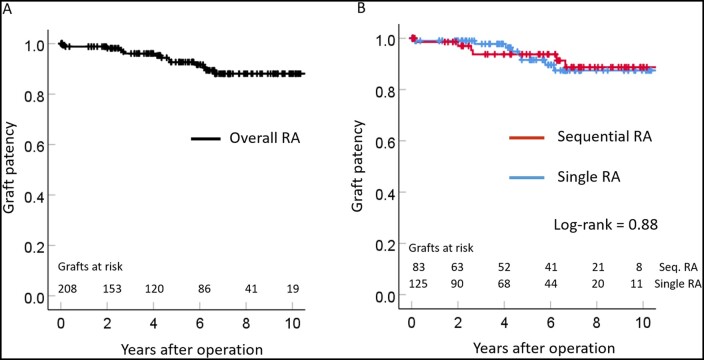
Kaplan–Meier graph showing graft patency rates for radial artery. (**A**) Overall and (**B**) sequential versus single. RA: radial artery; Seq: sequential.

**Figure 2:**
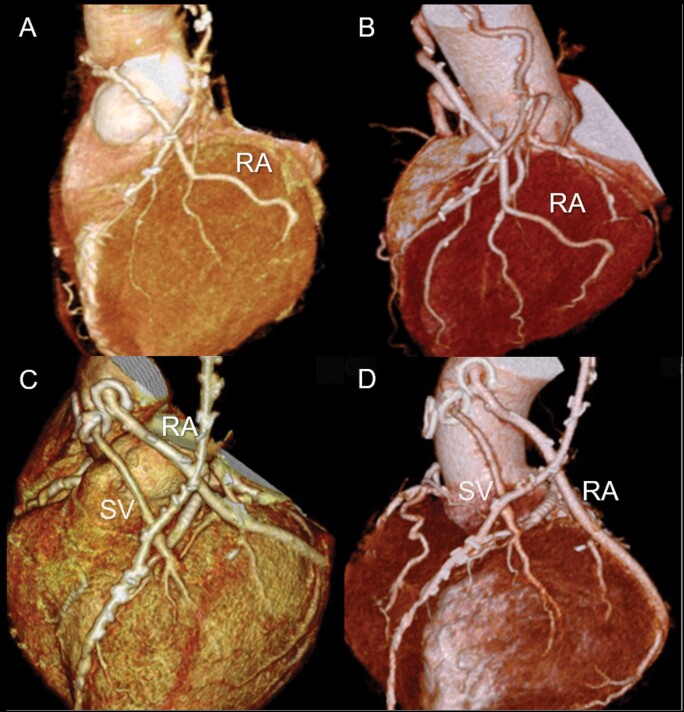
Surveillance computed tomography angiography. (**A **and** B**) A 70-year-old man who underwent coronary artery bypass grafting, including the sequential radial artery. Panel (**A**) is at one month, and panel (**B**) is 8 years after the operation. (**C **and** D**) A 60-year-old man who underwent coronary artery bypass grafting, including the sequential radial artery. Panel (**C**) is at 6 years and panel (**D**) is 12 years after the operation. RA: radial artery; SV: saphenous vein.

The 1-month and 1-, 5- and 10-year graft patency rates for the overall RA grafts were 99.4%, 99.4%, 92.7% and 88.1%, respectively (Fig. 1A); these did not differ from those of the ITA (100%, 100%, 97.3% and 88%, respectively; *P* = 0.62). The graft patency rates for sequential and single RA grafts were similar (*P* = 0.88, Fig 1B). Follow-up CT and invasive angiographic assessment revealed that the endoluminal outlines of all the patent RA grafts were smooth, without significant atherosclerotic changes (Fig. 2).

**Figure 3:**
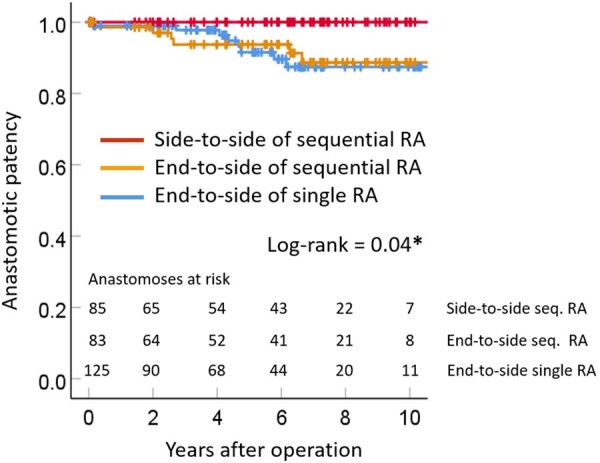
A comparison of the anastomotic patency rates of single radial artery grafts (blue line), the side-to-side anastomoses of sequential radial artery grafts (red line) and the end-to-side anastomoses of sequential radial artery grafts (yellow line). RA: radial artery.

In the stratified analysis for anastomotic patency, the 1-month and 1-, 5- and 10-year patency rates of end-to-side anastomoses for sequential RA grafting were 100%, 98.6%, 93.7% and 88.7%, respectively (Fig. 3). The 1-month and 1-, 5- and 10-year patency rates of end-to-side anastomoses for single RA grafting were 99%, 99%, 91.6% and 87.4%, respectively (Fig. 3). There was no significant difference regarding patency between end-to-side anastomoses for sequential and single RA grafting (*P* = 0.88). No late occlusion of side-to-side anastomoses for sequential RA grafting was observed; namely, the 10-year patency rate was 100% (Fig. 3). Therefore, the patency rate of side-to-side anastomoses of sequential RA grafting was significantly better than that of end-to-side anastomoses (*P* = 0.01). Finally, the anastomotic patency of side-to-side anastomoses for sequential RA grafting was significantly better than the patencies of end-to-side anastomoses of sequential and single RA grafting (*P* = 0.04, Fig. 3).

### Secondary analysis

**Figure 4:**
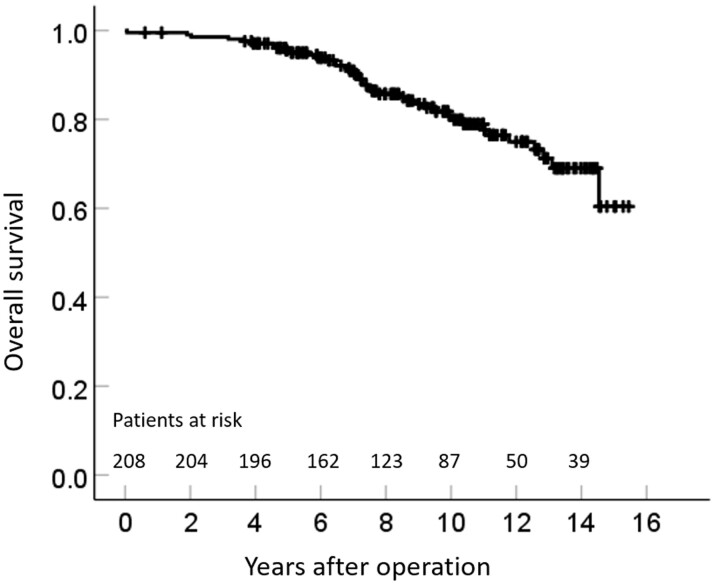
Kaplan–Meier curve of the overall survival after coronary artery bypass grafting using the radial artery (*n* = 208).

The median follow-up period was 9.1 years (interquartile range, 6.4–11.6) (range 0.1–15.4). Forty of the 208 patients died during the follow-up period. The estimated survival rate at 1, 5 and 10 years was 99.1%, 95% and 80.1%, respectively (Fig. 4).

### Lipid management

Statin therapy had been initiated after the diagnosis of coronary artery disease and was continued to reduce the LDL-C level to <100 mg/dl. Thereby, the mean LDL-C level of 107.8 ± 33.3 mg/dl measured just before surgery reduced to 90.3 ± 24.5 mg/dl at discharge and 92.9 ± 31.3 mg/dl at the end of the follow-up period. Only 4.3% of patients had LDL-C levels of 140 mg/dl or more at that time.

## DISCUSSION

Since Acar *et al.* [14] revived the RA as an arterial conduit for CABG, many encouraging findings regarding RA grafts have been reported. Barner *et al.* performed symptom-directed catheterization on 77 patients and reported that the free graft patency, including both single and sequential RA grafting, was 78.6% at 6.6 ± 4.0 years after CABG [15]. Possati *et al.* [16] reported that the long-term RA graft patency rate was 91.6%, confirmed by protocol-driven angiograms on a series of 90 consecutive patients at 8.8 ± 0.8 years postoperatively. Tatoulis *et al.* [17] performed symptom-driven angiography on 1108 patients, and the 5- and 7-year RA graft patency rates were 89% and 83%, respectively. In addition, they reported that RA grafts which had been patent on the prior angiograms maintained the smooth and uniform endoluminal appearances on the late angiograms [17]. Gaudino *et al.* [18] performed a prospective 20-year follow-up of 100 patients who had undergone RA grafting. They reported that the probability of RA graft failure was 25.0 ± 0.2% at 20 years after CABG. In the same series, 30 of the 100 patients underwent both 10- and 20-year angiographic assessments and the 10- and 20-year perfect RA patency rates without intraluminal irregularities were 86.6% and 73.3%, respectively [18]. Furthermore, in recent years, the results of comparative research on RA and other types of grafts (i.e. right ITA, SV) have been published. Such randomized evidence is increasingly encouraging the use of the RA graft for CABG [8, 9].

In the present study, the RAs were skeletonized and used mainly as second or third arterial conduits. The RA graft patency rate including both single and sequential grafting was 88.1% at 10 years (Fig. 1). This patency rate is comparable to the reported angiographic results. Moreover, these findings are similar to those previously reported, in that, almost all the patent RA grafts had no diffuse intraluminal irregularities suggesting severe atherosclerotic changes (Fig. 2D). Based on these findings, we believe that once RA grafts have obtained perfect patency, they can be expected to maintain long-term patency without rapid progression of atherosclerosis [19].

Sequential RA grafting is applied to increase the revascularization area by the arterial conduit. Reports regarding long-term sequential RA grafting are limited; however, Schwann *et al.* investigated symptom-driven angiographical results of sequential RA grafts in 122 patients at 2.7 ± 2.5 years postoperatively. Differences in graft patency between sequential and nonsequential grafts were not found likely due to the limited number of nonsequential RA grafts (175/252 [69%] vs 17/20 [85%]; *P* = 0.202). In addition, it was reported that anastomotic patencies of side-to-side and end-to-side RA anastomoses were identical (36 of 123 [29%]) vs (40 of 129 [31%]); *P* = 0.764) [20]. Hosono *et al.* recently reported a good 5-year RA graft patency rate of 86.5% confirmed by mainly MDCT angiography in 214 patients. In addition, the patency of sequential RA grafting was significantly better than that of single RA grafting; however, the side-to-side and end-to-side anastomotic patencies were not compared [21]. In our study, long-term distal end-to-side anastomotic patencies of single and sequential RA grafting were equally high (87.4% vs 88.7% at 10 years) and the anastomotic patency rate of side-to-side anastomoses for sequential RA grafting was 100%. This patency rate was significantly better than that for the distal end-to-side anastomosis. Gaudino *et al.* [22] reported that the RA grafts obtained from the distal portion showed higher vasospastic tendencies and more string signs, and the mid-term perfect patency was lower when compared with RA grafts harvested from the proximal portion. Their findings may be influenced by the properties of the RA. The distal portion of the RA generally has a smaller diameter, and the muscular component of the media is thicker than that of its proximal portion. We harvested the RAs with the skeletonization technique to lengthen the graft and avoid using the distal part of the RA as much as possible (see Supplementary Material, Fig. S1) [12]. Our grafting technique limited the number of side-to-side anastomoses to one, at least in principle. The distal end of the RA graft was anastomosed to a target vessel with good run-off, and the RA graft was usually placed perpendicularly to the target vessels to save the graft length. Our sequential RA grafting using a single side-to-side anastomosis limited the revascularization area compared with RA grafting with multiple side-to-side anastomoses; however, our good long-term anastomotic patencies could be related to our grafting strategy. Further investigations are needed to evaluate the effective and efficient use of the RA.

We asked family doctors to care for the patients after discharge from the hospital, according to societal guidelines [23]. As a result, postoperative LDL-C levels were maintained close to the target values in most patients. Diltiazem hydrochloride or nicorandil was administered unless contraindicated in order to prevent RA vasospasm in this series. However, it is yet to be determined whether the administration of calcium blockers improves the mid-term RA graft patency [24]. We believe that our postoperative management was responsible for the good long-term patency and the low rate of major adverse cardiac events.

### Limitations 

This study is subject to the limitations inherent to retrospective observational designs. Although patient characteristics were almost comparable between the groups, there was no matching of patients based on clinical and demographic characteristics due to the relatively small number of included participants. There may be patient, graft or target coronary selection biases for RA grafting. For example, dialysis patients were excluded to use the RA and the number of females who underwent sequential RA grafting was significantly lower than that of males. Moreover, the inclusion of various factors that potentially affect the long-term graft patency, such as RA harvesting technique and use of off-pump bypass grafting, may limit the results of this study. However, the overall RA patency rate is nevertheless close to the ITA patency rate in this study; therefore, we believe that the RA is an attractive arterial conduit that can be used as a second or third arterial graft. Sequential RA grafting for selected non-LAD targets is also attractive because of the high anastomotic patency of side-to-side anastomoses.

## CONCLUSION

The long-term graft patencies of both the single and sequential RA grafts for second or third arterial conduits were equally high. The side-to-side anastomotic patency of sequential RA grafts with a single side-to-side anastomosis was prominently higher than both the distal end-to-side anastomotic patencies of sequential and single RA grafting. Taking these results into account, the RA can be effectively used for arterial coronary revascularization.

## SUPPLEMENTARY MATERIAL

Supplementary material is available at *ICVTS* online.

## Supplementary Material

ivab279_Supplementary_Data

## ABBREVIATIONS

CABG: Coronary artery bypass grafting
ITA: Internal thoracic artery
LAD: Left anterior descending artery
LDL-C: Low-density lipoprotein cholesterol
MDCT: Multi-detector computed tomography
RA: Radial artery
SV: Saphenous vein
