# Supplementary material for: Comparison of patency of single and sequential radial artery grafting in coronary artery bypass
Source: Interact Cardiovasc Thorac Surg. 2021 Oct 23;34(4):515–22. doi: 10.1093/icvts/ivab279 (PMC8972210; doi:10.1093/icvts/ivab279)
Supplement: ivab279_Supplementary_Data [file ivab279_supplementary_data.docx]

**Supplementary Material**

**Supplement Fig. S1.** The radial arteries were harvested with the skeletonization technique to lengthen the graft.


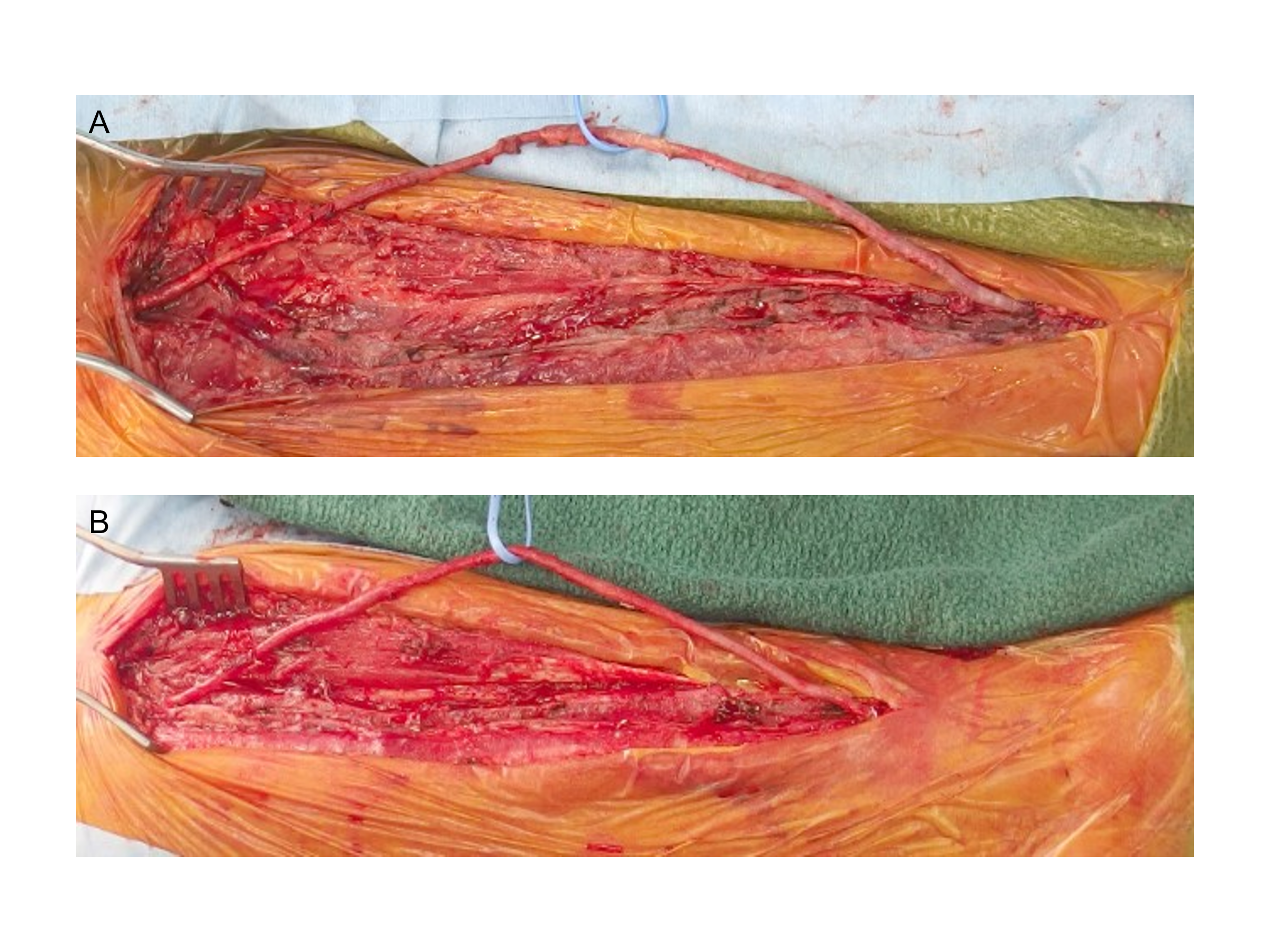


A: A vessel loop was placed around the radial artery loosely without tension. This patient’s height was 158 cm. The length of the radial artery was sufficient for sequential grafting of the circumflex arteries.

B: Intraoperative photograph of a patient who was 162 cm in height. A vessel loop was placed around the radial artery. The incision was not made in the vicinity of the wrist.

**Supplement Table S1** Distal anastomotic sites by each graft

|  | RCA | LAD | D, IM | LCx |
| --- | --- | --- | --- | --- |
| ITA | 4 | 204 | 24 | 14 |
| Left |  | 188 | 20 | 11 |
| Right | 4 | 16 | 4 | 3 |
| RA | 32 | 11 | 99 | 151 |
| SV | 199 |  | 18 | 65 |
| GEA | 1 |  |  |  |

The number of distal anastomoses is presented in this table.

Abbreviations: D, diagonal; GEA, gastroepiploic artery; IM, intermediate; ITA, internal thoracic artery (left and right sides); LAD, left anterior descending artery; LCx, left circumflex; RA, radial artery; RCA, right coronary artery; SV, saphenous vein.
